# 407. Minimum Manufacturing Costs, National Prices and Estimated Global Availability of New Repurposed Therapies for COVID-19

**DOI:** 10.1093/ofid/ofab466.608

**Published:** 2021-12-04

**Authors:** Junzheng Wang, Jacob Levi, Leah Ellis, Andrew Hill

**Affiliations:** 1 Imperial College, London, England, United Kingdom; 2 University College London Hospitals, London, England, United Kingdom; 3 Imperial College London, London, England, United Kingdom; 4 University of Liverpool, London, England, United Kingdom

## Abstract

**Background:**

Currently, only dexamethasone, tocilizumab and sarilumab have conclusively been shown to reduce mortality of COVID-19. No drug for prevention or treatment in earlier stages of COVID-19 are yet found, with previously promising drugs such as hydroxychloroquine and remdesivir have been shown to be ineffective. Several new candidates are now being studied in clinical trials. Safe and effective treatments will need to be both affordable and widely available. We therefore revised our original 2020 analysis to reflect recent developments. In this update we analysed the cost of production, current national list prices, and API availability for oral and IV dexamethasone, ivermectin, colchicine, dutasteride, budesonide, baricitinib and monoclonal antibodies tocilizumab and sarilumab.

**Methods:**

Costs of production for new and potential COVID-19 drugs (dexamethasone, ivermectin, dutasteride, budesonide, baricitinib, tocilizumab, sarilumab and colchicine) were estimated using an established and published methodology based on costs of active pharmaceutical ingredients (API), extracted from the global shipping records database Panjiva. This was compared with national pricing data from low, medium, and high-income countries. Annual API export volumes from India were used to estimate the current availability of each drug.

**Results:**

Repurposed therapies can be generically manufactured at very low per-course costs: ranging from &2.58 for IV dexamethasone (or &0.19 orally) to &0.12 for ivermectin. No export price data was available for baricitinib, tocilizumab or sarilumab. When compared against international list prices, we found wide variations between countries. Drug API availability was generally good, with colchicine being the most available with sufficient annual API exported for 59.8 million treatment courses. A summary is shown in Table 1.

Table 1. Summary of list prices, estimated production costs, and current availability of potential COVID-19 drugs selected for analysis. OD = Once daily, BD = twice per day, EUA = Emergency Use Authorisation (only to be given with remdesivir) *In most recent 12-month period.

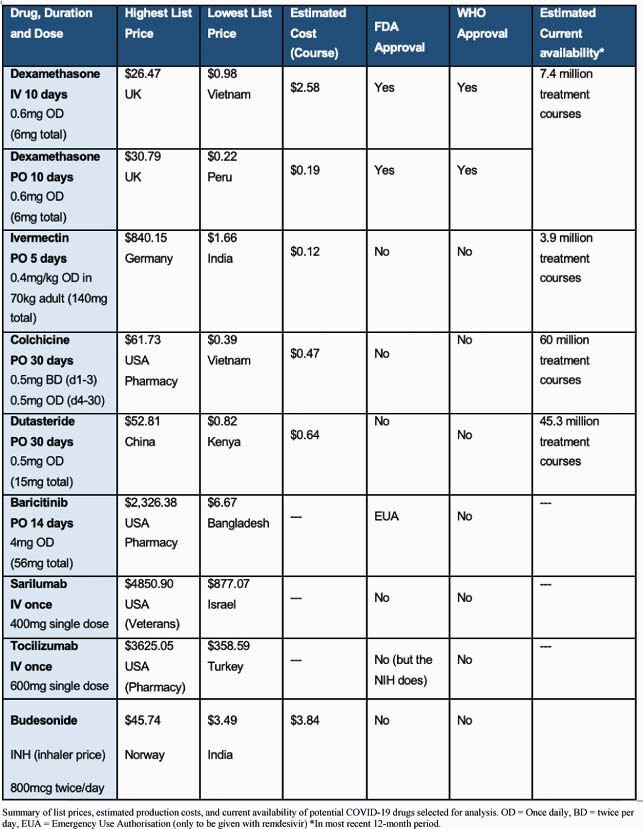

**Conclusion:**

Successful management of COVID-19 will require equitable access to treatment for all, not just those able to pay. Repurposed drugs can be manufactured at very low costs if shown to be clinically effective, and offers an affordable, widely available option for patients at all stages of the disease from pre-exposure prophylaxis to asymptotic and mild infections, through to critical care until vaccination coverage is expanded.

**Disclosures:**

**All Authors**: No reported disclosures

